# The Eeffect of Metformin Combined with Calcium-Vitamin D_3_ Against Diet-Induced Nonalcoholic Fatty Liver Disease

**DOI:** 10.15171/apb.2018.012

**Published:** 2018-03-18

**Authors:** Sara Shojaei Zarghani, Samin Abbaszadeh, Mohammad Alizadeh, Maryam Rameshrad, Alireza Garjani, Hamid Soraya

**Affiliations:** ^1^Student Research Committee, Urmia University of Medical Sciences, Urmia, Iran.; ^2^Cellular and Molecular Research Center, Department of Pharmacology, Faculty of Pharmacy, Urmia University of Medical Sciences, Urmia, Iran.; ^3^Department of Nutrition, Food and Beverages Safety Research Center, Faculty of Medicine, Urmia University of Medical Sciences, Urmia, Iran.; ^4^Department of Pharmacology and Toxicology, Faculty of Pharmacy, Tabriz University of Medical Sciences, Tabriz, Iran.

**Keywords:** AMP-activated protein kinase, Calcium, Metformin, Nonalcoholic fatty liver disease, Vitamin D_3_

## Abstract

***Purpose:*** Metformin is one of the most popular drugs tested against nonalcoholic fatty liver disease (NAFLD). The present study aimed to investigate whether calcium-vitamin D_3_ cosupplementation will intensify the effect of metformin on the prevention of high-fat, high-fructose (HFFr) diet-induced hepatic steatosis.

***Methods:*** Male wistar rats (210±16 g) were assigned into the following seven groups: a Control group to receive a standard chow and six HFFr-fed groups to receive diets containing either normal (0.5% calcium and 1000 IU/kg vitamin D_3_) or high amount of calcium and vitamin D_3_ (2.4% calcium and 10000 IU/kg vitamin D_3_) (CaD), in combination with gastric gavage administration of either saline or 25 or 200 mg/kg body weight/day metformin. After 60 days, rats were assessed with respect to their anthropometric, metabolic and hepatic parameters, as well as their hepatic AMP-activated protein kinase (AMPK) phosphorylation.

***Results:*** Metformin and CaD, either alone or in combination, caused a significant reduction in HFFr diet-induced high serum aspartate aminotransferase (AST), hepatic steatosis and lipid accumulation without effect on insulin resistance and AMPK phosphorylation. In addition, slightly (and non-significantly) better effects of the combination in ameliorating steatosis and hepatic cholesterol content were observed.

***Conclusion:*** Taken together, our results suggest that metformin and CaD could protect against the onset of HFFr diet-induced NAFLD in an insulin and AMPK-independent manner, without any marked additional benefits of their combination.

## Introduction

Nonalcoholic fatty liver disease (NAFLD) has become the most common form of chronic liver disease in the world, which is characterized by abnormal triglyceride accumulation in hepatocytes, not due to excess alcohol consumption or other causes of secondary hepatic steatosis.^1,2^ A growing body of evidence suggests a bidirectional relationship between NAFLD and components of metabolic syndrome,^3,4^ although insulin resistance may still play an important role in the pathogenesis of NAFLD.^5^ It is believed that insulin resistance and adiposity are associated with an imbalance between delivery and export of free fatty acid to the liver.^1,5^ Therefore,‏ it is not surprising that several studies have investigated the‏ eﬃcacy of natural or pharmacological insulin sensitizers on the prevention and management of NAFLD.

Among pharmacological‏ insulin sensitizers, metformin has acquired a fundamental‏ role in the management of many disorders associated‏ with insulin resistance. Metformin is believed to exert its anti-diabetic effects through inhibition of mitochondrial respiratory chain complex I,^6,7^ resulting in a fall in the ATP/AMP ratio and activation of AMP-activated protein kinase (AMPK), a master kinase regulating cellular energy homeostasis.^8^ The activation of AMPK in the liver could both induce catabolic pathways, such as β-oxidation of fatty acids, and suppress anabolic pathways like lipogenesis, thus potentially leading to reduced hepatic steatosis.^8,9^ As some studies have shown that metformin could protect against the development of steatosis in animal models,^10,11^ however human studies are controversial.^12^

There is now some evidence that vitamin D could be a natural insulin sensitizer, especially in combination with calcium.^13,14^ Dietary calcium or VitD_3_ (cholecalciferol) intake, as well as calcitriol administration have been shown to prevent adiposity, insulin resistance^14,15^ and hepatic fatty changes^16,17^ and to be related to AMPK activation in different tissues in animal models.^18,19^ On the other hand, Shalata *et al*.^20^ reported that vitamin D could intensify the effects of sitaglibtin/metformin drugs on treatment of steatosis and on decreasing hepatic triglyceride and malondialdehyde (MDA) content. So the hypothesis that combined calcium and vitamin D_3_ supplementation augments the protective effects of metformin on the onset of diet-induced steatosis was tested in the present study, and the role of AMPK signaling in mediating observed effects was also determined.

## Materials and Methods

### Animals and diets

Male Wistar rats (210±16 g) were housed at a room temperature of 22±2°C under a 12-h light–dark cycle. The rats were acclimated to the new conditions for one week before receiving their experimental diets. Then, rats were randomly assigned to two groups to feed on either a standard chow (Control, n=6) or high-fat, high-fructose (HFFr) diet (n=42) for a period of 60 days. Rats had continuous access either to plain tap water or to water containing 20% fructose solution, respectively. The HFFr-fed rats were nourished ad libitum with a normal calcium (0.5%) and VitD_3_ (1000 IU/kg) diet or a high calcium (2.4%) and VitD_3_ (10000 IU/kg) diet (CaD). These rats were also randomized to receive, by gastric gavage,* either* 1 ml of saline alone *(S) or 25 or 200 mg/kg body weight metformin,* once a day. The standard chow contained recommended levels of calcium (0.5%) and VitD_3_ (1000 IU/kg).^21^*The highest amount of vitamin* D_3_*(10000 IU/kg diet)* was selected as the optimal level of vitamin D_3_ intake without any adverse effect on rats.^22^ Details of Control and HFFr diets are provided in Table 1. The HFFr diet used has been shown to induce NAFLD, previously.^23^ Throughout the experiment, daily feed intake and weekly body weight measurements were taken to monitor the health of the rats. This study was conducted in accordance with the Guide for the Care and Use of Laboratory Animals of Urmia University of Medical Sciences. In addition, all animal protocols were approved by the Ethics Committee of Urmia Medical Sciences University.

**Table 1:** Macronutrient composition and energy contents of the control and high fat high fructose (HFFr) diets

**-**	**Constituent**	**Control**	**HFFr**
Macronutrients (% by weight)	**Carbohydrate**	52.8	47.9
	*Starch*	52.8	30.4
	*Fructose*	0	17.5
	**Fat**	5	27.8
	*Soybean oil*	5	2.8
	*Hydrogenated oil*	0	5
	*Sheep tallow*	0	20
	**Protein**	20	11.5
Macronutrients (% Kcal)	**Carbohydrate**	59.7	37.3
	**Fat**	14.5	53
	**Protein**	25.8	9.7
	Energy (Kcal/g)	3.1	4.72

### Evaluation of animal and organ weights

At the end of the 60^th^ day of the diet, the animals were anesthetized by an *ip* injection of a mixture of ketamin (60 mg/kg) and xylazin (10 mg/kg). Then, after blood sampling, the livers and visceral fat from both epididymal and perirenal areas were rapidly excised and weighed. Next, the livers were stored at -80 for further analysis.

### Serum analysis 

At the end of the treatment period and after a 16 h fast with free access to water, blood samples were collected via portal vein from anaesthetized animals. Blood samples were centrifuged and serum was frozen at -80°C for subsequent analysis. Serum fasting glucose, alanine aminotransferase (ALT), aspartate aminotransferase (AST), triglycerides, total and high-density lipoprotein (HDL) cholesterol concentrations were determined by an enzymatic method using individual commercial kits (Pars azmun, Tehran, Iran) and an automatic biochemical analyzer (BT 4500, Biotechnica, Italy). Non-HDL-C was calculated as total cholesterol minus HDL-C. Serum insulin (Bioassay Technology Laboratory, China) and complement C1q/tumor necrosis factor-α related protein-3 (CTRP3) (zellBio, Germany) levels were quantiﬁed with ELISA kits. In addition, we used the homeostasis model assessment of insulin resistance (HOMA-IR) method to estimate insulin resistance as [Fasting insulin×Fasting glucose/405].

### Hepatic lipid quantitation and lipid peroxidation

The hepatic lipids were extracted according to the method described by Folch *et al*.^24^ Hepatic triglyceride and cholesterol content was quantified using commercially colorimetric assay kits (Pars azmun, Iran). Results were given as mg triglyceride or cholesterol per gram liver. Hepatic malondialdehyde (MDA), a product of lipid peroxidation, was also measured to evaluate the degree of oxidative stress, as described by Yousefi *et al.*^25^

### Histological examination

Immediately after removal, fragments of liver tissues were ﬁxed in a solution of 10% buffered formaldehyde. Sections of the formalin-fixed and paraffin-embedded tissues were stained with hematoxylin and eosin to semi-quantitatively assess the fatty degeneration using the NAFLD activity score (NAS). The histological features were numerically scored according to the percentage of distributions and blinded regarding treatment groups. Scores for steatosis (score 0 to 3), lobular inﬂammation (score 0 to 3), and ballooning (score 0 to 2), were also summed to produce the NAS, thus they ranged from 0 to 8.

### Western blot analysis

Powdered liver tissue, was homogenized (10% w/v) in homogenization buffer containing 50 mM Tris-Hcl, 150 mM Nacl, 5mM Sodium Pyrophosphate (NaPPi), 50mM NaF, 1mM EDTA, 1mM dithiothreitol (DTT), 0.1%SDS (w/v), 1% TXT-100 (v/v), and protease inhibitor cocktail. Tissue homogenates were centrifuged at 1000g for 10 min at 4 °C and supernatants were stored in 50 µl aliquots at - 80 °C for further analysis. Bradford Protein Assay kit was used to evaluate the protein content of the supernatant. SDS-polyacrylamide gel electrophoresis, followed by transfer to nitrocellulose membranes was performed using 50 μg of homogenate protein. Then membranes were blocked in 5% non-fat milk in Tris-buffered saline Tween-20 and incubated overnight with rabbit antibodies against phospho-AMPK (p-AMPKThr172) and AMPK (1:1000 dilution - Cell Signaling Technology Inc., Danvers, Massachusetts, USA) in 5 % BSA (wt/vol). After extensive washing, the membranes were incubated with a peroxidase-conjugated goat anti-rabbit secondary antibody (1:5000 dilution, Cell Signaling Technology Inc. Danvers, Massachusetts, USA) in 5% skim milk (wt/vol). After washing, antibodies were visualized using the BM Chemiluminescence kit (Roche; Germany). Densitometric analyses of immunoblots were performed using Image J software (National Institute of Health, Bethesda, Maryland).^26^

### Statistical analysis

All data are presented as mean ± standard error (SE). Data were tested by one-way analysis of variance (ANOVA, SPSS19). When ANOVA results were significant (p<0.05), the post hoc Tukey test was also applied to find where differences existed.

## Results and Discussion

### Body and Tissue Weights

The initial body weight of rats was similar (*p-value*=0.19). At the end of the experimental period, the HFFr+S rats had a near doubling of visceral fat weight compared to the Control group (6.9±1.1 g vs. 3.4±0.4 g, *p-value*=0.009), while it was slightly and non-significantly lower in the other groups, especially in the rats gavaged with the high dose of metformin (200 mg/kg/day). After 60 days, no significant differences were found between the groups regarding final body weight and liver/body weight percentage (Table 2). It means that the excess energy intake of HFFr rats have led to a greater adiposity, but not to a higher body weight.

**Table 2:** Body and tissue weight-related measurements

-	**Control**	**HFFr+S**	**+Met25**	**+CaD+Met25**	**+Met200**	**+CaD+Met200**	**+CaD+S**
Initial Body Weight (g)	213.3±6.5	218.7±6.8	208±5.6	203±3.9	201.6±6	203±5.4	221.1±9
Final Body Weight (g)	288.3±13.6	278.8±11.1	243.7±9.4	255.1±7	238.7±9.5	244.5±13.1	262.3±6.5
Liver/ body weight (%)	3.02±0.05	2.9±0.05	3±0.07	3.1±0.09	3.1±.1	3.1±0.09	2.9±0.1
Visceral fat weight (g)	3.4±0.4	6.9±1.1^*^	5.5±0.6	5.1±0.3	4.6±0.5	4.7±0.3	5.1±0.5
Visceral fat/body weight (%)	1.1±0.13	2.4±0.3^*^	2.3±0.2	2.01±0.14	1.91±0.14	2.02±0.14	1.94±0.2

HFFr: High-Fat, High-Fructose diet, S: Saline, +Met25: HFFr+25 mg/kg body weight metformin, +Met200: HFFr+200 mg/kg body weight metformin, +CaD: HFFr+2.4% Calcium plus 10000 IU/kg vitamin D_3_. Data are mean ± SE. n = 6–7 in each group. (*) P<0.05 versus the Control group. (one way ANOVA, Tukey post hoc test).

### Serum Biochemistry

Compared to the Control rats, the HFFr+S rats displayed a non-significant increase in glucose levels and HOMA-IR scores and a decrease in insulin levels. The CaD+S rats had also greater glucose and lower insulin levels compared to the metformin-gavaged rats (not significantly in some cases), although no meaningful differences were observed in HOMA-IR between the groups (*p-value*=0.34).

Vitamin D and calcium have been linked to the improvement of insulin sensitivity, activation of the Ca^2+^ -mediated apoptotic pathway in adipose tissues, ^14^ inhibition of lipogenesis and promotion of lipid oxidation. ^17,27^ The beneficial effects of metformin are also thought to be through some mechanisms, including activation of AMPK, ^28^ and increasing insulin receptor activation, glucose uptake ^29^ and leptin sensitivity in liver. ^30^ In the present study, we tested the hypothesis that whether or not CaD augments the ameliorating effects of metformin on insulin resistance, as the major pathogenic factor of NAFLD. Surprisingly, to the contrary of our hypothesis, neither metformin nor CaD did not alter the increased levels of HOMA-IR induced by HFFr diet, so exerted their protective effects independent of insulin action. The inability of metformin therapy to reduce indexes of insulin resistance has been reported in both humans^31,32^and animal studies,^10,33^ that in part may be due to the unaltered insulin signaling within skeletal muscle and whole-body, and gluconeogenesis after metformin treatment.^31,34^ In this line, Linden *et al*.^35,36^ reported that metformin (300 mg/kg/day) lowered adiposity, hemoglobin A1c levels, hepatic triglycerides and markers of hepatic de novo lipogenesis in diabetic Otsuka Long-Evans Tokushima Fatty (OLETF) rats, which were not accompanied by an improvement in the both fasting and postchallenge glycemic control and in the increased serum levels of triglyceride and free fatty acids. Indeed, there are some other studies which have found no effect exerted by either dietary calcium and/or vitamin D on glucose intolerance.^37-41^

In this study, HFFr diet, calcium-vitamin D_3_ supplementation and oral gavage administration of metformin did not alter the serum concentrations of triglycerides, total cholesterol and Non-HDL-C (*p-value*>0.05). The rats in the CaD+S group exhibited clearly higher levels of serum HDL-C compared to the groups receiving recommended levels of calcium and vitamin D_3_. Moreover, the levels of serum CTRP3 was significantly higher in the +Met200 group compared to the other groups (except the +CaD+Met200 group). There was *no effect of HFFr diet and no* additive effect of metformin and CaD on serum HDL-C and CTRP3 (Table 3).

**Table 3:** Serum Biochemical Parameters

-	**Control**	**HFFr+S**	**+Met25**	**+CaD+Met25**	**+Met200**	**+CaD+Met200**	**+CaD+S**
Triglycerides (mg/dl)	49.3±3.8	41.2±4.1	35.1±2.7	48.5±2.9	44.2±5.2	45.8±6.2	38±6.8
Cholesterol (mg/dl)	63±4.7	59.6±1	66±3.8	64.2±4.5	56.1±4.1	62.6±2.3	65.8±3.8
HDL-C (mg/dl)	20±0.9	20.3±0.4^a^	22.1±1.1^a,c^	23.3±0.7^a,b^	22.2±1.4^a,c^	26±0.8^b,c^	26.6±0.8^b^
Non-HDL-C (mg/dl)	41.7±4.2	39.6±1.4	43.8±3.1	44.1±3.3	33.8±3.6	36.6±2.1	39.1±4.6
Glucose (mg/dl)	114±8.3	170.5±9^a,b^	135±16.2^a^	152.8±19.9^a,b^	167.2±20.7^a,b^	154.5±14.9^a,b^	216.8±5.8^b^
Insulin (mg/dl)	13.01±0.9	12.4±0.3^a,b^	13.4±0.7^a^	13.2±0.5^a^	12.1±0.8^a,b^	14.4±0.4^a^	10.38±0.5^b^
HOMA-IR	3.8±0.4	5.2±0.3	4.9±0.4	5.4±0.8	4.9±0.6	5.5±0.6	5.3±0.2
CTRP3 (ng/ml)	128.4±18.1	127.6±22.3^a^	131.8±10^a^	140.3±16.1^a^	235.6±12.9^b^	168.2±21.4^a,b^	98.1±16.6^a^

HFFr: High-Fat, High-Fructose diet, S: Saline, +Met25: HFFr+25 mg/kg body weight metformin, +Met200: HFFr+200 mg/kg body weight metformin, +CaD: HFFr+2.4% Calcium plus 10000 IU/kg vitamin D_3_, HDL-C: High-density lipoprotein cholesterol, HOMA-IR: Homeostatic model assessment of insulin resistance, CTRP3: Complement C1q/tumor necrosis factor-α related protein-3. Data are mean ± SE. n = 6–7 in each group. (*) P<0.05 versus the Control group. Values with different letters are significantly different (P<0.05) (one way ANOVA, Tukey post hoc test).

CTRP3 (also known as cartonectin, cartducin, CORS-26) is a novel adipokine and a member of CTRP superfamily, which has been shown to reduce glucose levels, hepatic steatosis and gluconeogenesis, by its regulatory effects on lipid and glucose metabolism.^42,43^ Moreover recent studies have suggested that some beneficial effects of CTRP3 could be mediated through AMPK signaling.^44-46^ The reduced levels of circulating and tissue expression of CTRP3 are reported in both human and rodent models of obesity and diabetes.^43,47-49^ Nonetheless, choi *et al*.^50^ reported an elevated circulating CTRP-3 concentrations in diabetic compared to non-diabetic adults and in the another study no difference was found between patients with and without metabolic syndrome.^51^ In the present study, although neither the HFFr diet nor CaD supplementation did not alter the circulating levels of this adipokine, metformin administration at a dose of 200 mg/kg led to the highest levels of CTRP3, but not the most improvement in AMPK activation and steatosis. This was, in part, in accordance with *findings of* Tan and coworkers^52^ who for the first time investigated the effects of metformin therapy on CTRP3 levels. They demonstrated lower serum and omental adipose tissue CTRP3 in women with PCOS, which was increased after 6 months of metformin therapy.

**Figure 1:**
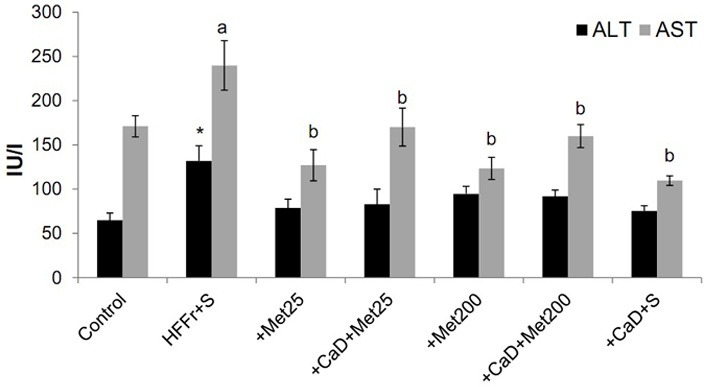
Effect of experimental diets on liver enzymes. HFFr: High-Fat, High-Fructose diet, S: Saline, +Met25: HFFr+25 mg/kg body weight metformin, +Met200: HFFr+200 mg/kg body weight metformin, +CaD: HFFr+2.4% Calcium plus 10000 IU/kg vitamin D_3_, ALT: Alanine aminotransferase, AST: Aspartate aminotransferase. Data are mean ± SE. n = 6–7 in each group. (*) P<0.05 versus the Control group. Values with different letters are significantly different (P<0.05) (one way ANOVA, Tukey post hoc test).

Moreover, in the present study, the HFFr+S rats showed higher levels of serum ALT and AST compared to the Control group (131.9±17.6 IU/l vs. 65.2±8.1 IU/l, *p-value=* 0.02 and 239.8±27.9 IU/l vs. 171.5±12 IU/l, *p-value=*0.12, respectively). CaD supplementation and oral gavage of metformin significantly prevented the increase in the serum concentration of AST, although ALT levels reduced non-significantly, without any differences between groups. (Figure 1).

### Hepatic lipid content and lipid peroxidation

The HFFr+S rats exhibited the expected increases in the hepatic fat deposition (hepatic triglyceride, 5.75±0.59 mg/g tissue weight vs. 1.83±0.25 mg/g tissue weight, *p-value<* 0.001 and hepatic cholesterol 1.2±0.13 mg/g tissue weight vs. 0.88±0.06 mg/g tissue weight, *p-value=*0.04, respectively), while it was signiﬁcantly attenuated in the other groups. There was no marked differences in the hepatic triglyceride and cholesterol content between CaD-supplemented and/or metformin-gavaged groups.

**Figure 2:**
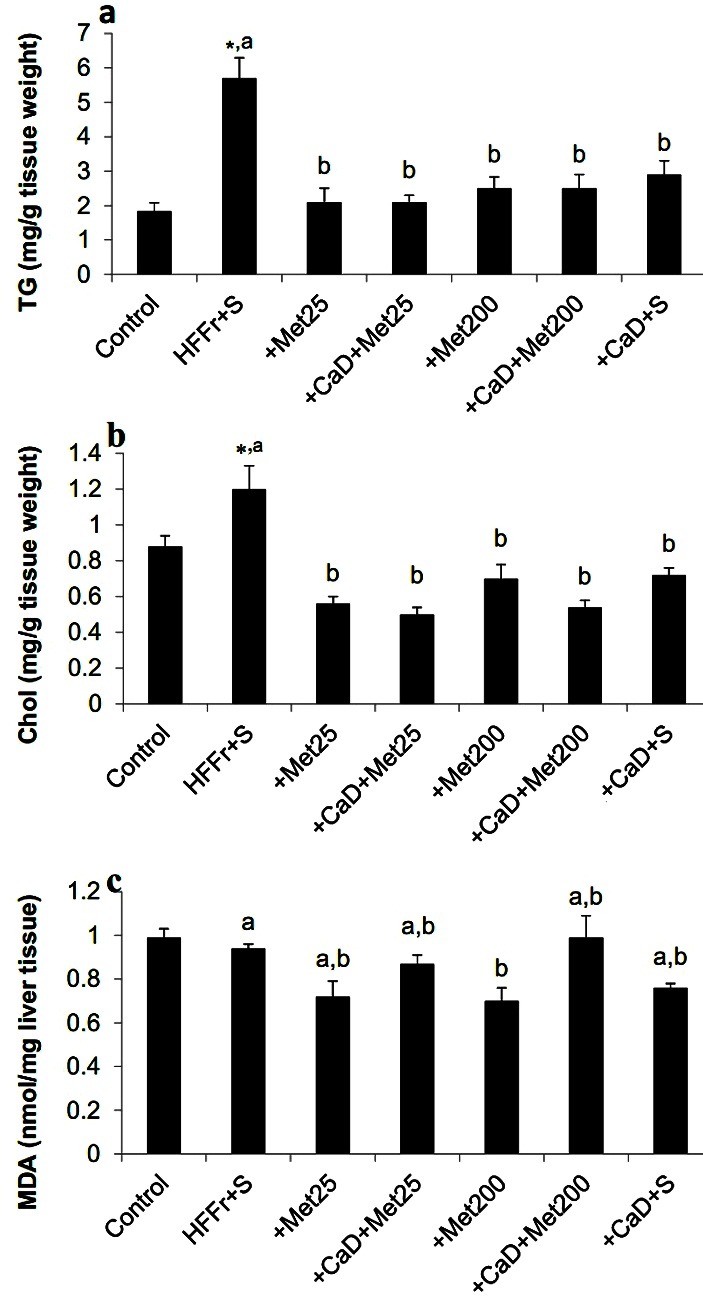
Effect of experimental diets on hepatic lipid content and malondialdehyde. **a** hepatic TG, **b** hepatic Chol, **c** hepatic MDA content. HFFr: High-Fat, High-Fructose diet, S: Saline, +Met25: HFFr+25 mg/kg body weight metformin, +Met200: HFFr+200 mg/kg body weight metformin, +CaD: HFFr+2.4% Calcium plus 10000 IU/kg vitamin D_3_, TG: Triglyceride, Chol: Cholesterol, MDA: Malondialdehyde. Data are mean ± SE.
n = 6–7 in each group. (*) P<0.05 versus the Control group. Values with different letters are significantly different (P<0.05) (one way ANOVA, Tukey post hoc test).

HFFr diet did not increase the lipid peroxidation in livers, which was determined by measurement of hepatic MDA contents, although the concentration of hepatic MDA was signiﬁcantly decreased in the +Met200 group compared to the HFFr+S group (Figure 2).

### Liver Histology

**Figure 3:**
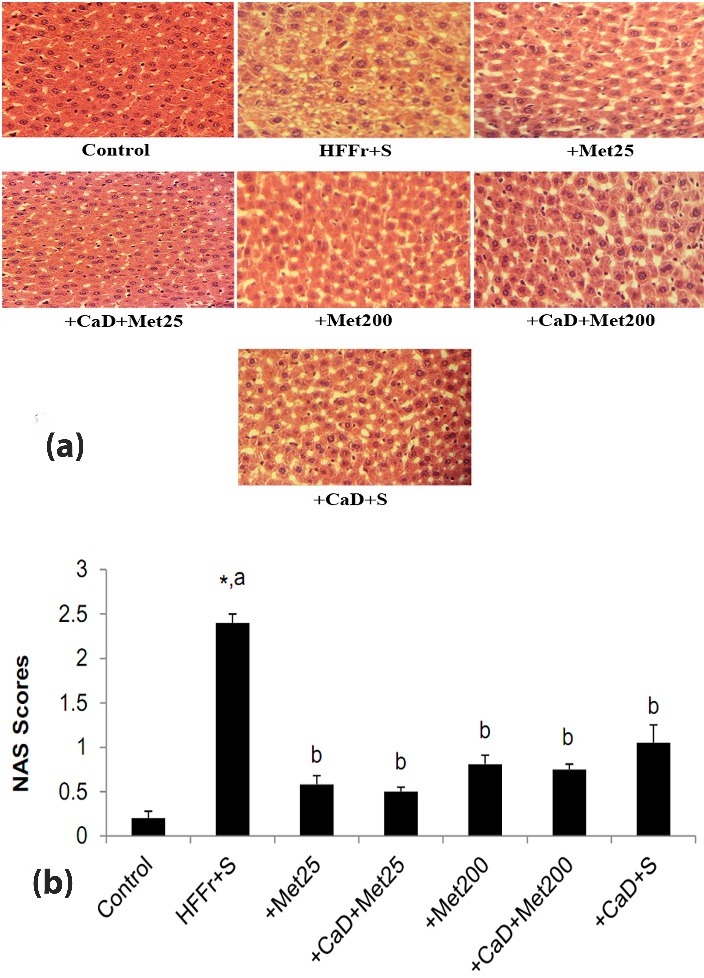
**a**: Histopathological assessment after haematoxylin–eosin staining of liver sections. Sections from liver tissues of the HFFr+S rats showed hepatic steatosis and only slightly ballooning degeneration. Other five groups, especially the metformin-gavaged groups, showed lower fat accumulation (magnification×400). **b**: The NAFLD activity score (NAS) was determined based on histopathological analysis (steatosis, inflammation and ballooning). HFFr: High-Fat, High-Fructose diet, S: Saline, +Met25: HFFr+25 mg/kg body weight metformin, +Met200: HFFr+200 mg/kg body weight metformin, +CaD: HFFr+2.4% Calcium plus 10000 IU/kg vitamin D_3_. Data are mean ± SE. n = 6–7 in each group. (*) P<0.05 versus the Control group. Values with different letters are significantly different (P<0.05) (one way ANOVA, Tukey post hoc test).

The livers from HFFr+S rats displayed evidence of fatty liver, with macro and microvesicular steatosis. Although steatosis and NAS scores were significantly attenuated in the other five groups, especially in the metformin groups, compared to that in the HFFr+S group, in accordance with the hepatic triglyceride content. The hepatic histological examination of liver fragments showed no sign of inﬂammation, and only *slightly ballooning* degeneration, as is shown in Figure 3.

Excessive fat accumulation in hepatocytes is the earliest and *the most common* manifestation of NAFLD.^53^*In the present study, although liver weight did not differ between groups, serum markers of liver function, hepatic lipid content and steatosis scores were raised in the HFFr+S rats and similarly attenuated in the treated groups in an insulin-independent manner. However, metformin, especially in the dose of 25 mg/kg/day, showed slightly more favorable effects on steatosis and hepatic lipid content. Our present results demonstrated that* using the combination of metformin and CaD, was no more effective than each of them alone on the different parameters studied. Although they *slightly* intensified the hepatoprotective effects of each other on HFFr diet-induced steatosis and hepatic cholesterol accumulation, but not on the increased serum liver enzymes levels and hepatic lipid peroxidation. *These results are consistent with the findings of some earlier* studies which have demonstrated the protective effects of each of metformin,^10,11^ calcium^16^ or 1,25 (OH)_2_ vitamin D_3,_^17^ separately, and CaD^54^ against the development of steatosis in animal models.

Calcium-vitamin D has been shown to increase the effects of metformin therapy on symptoms of women with polycystic ovary syndrome (PCOS).^55,56^ Nevertheless, according to the best of our knowledge, no report exists on the effect of combined calcium-vitamin D and metformin on the development of diet-induced NAFLD. Shalata* et al. reported that* vitamin D could intensify the effects of sitaglibtin/metformin drugs on treatment of steatosis.^20^ Moreover, a very recent study demonstrated the synergistic protective effects of vitamin D (6 ng/kg SC) and metformin (100 mg/kg) on the improvement of some metabolic and structural abnormalities and, hepatic steatosis induced by 8 weeks of high fat diet feeding in wistar rats.^57^ In this study no possible mechanism was mentioned for the observed synergistic effects, except a speculation of the involvement of AMPK.

### Western blot analysis

**Figure 4:**
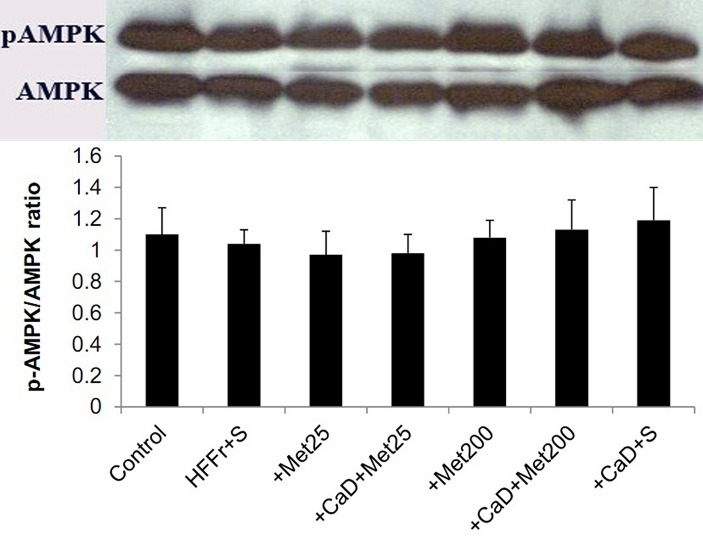
Western blotting analysis of AMPK and p-AMPK expression in the livers of experimental rats. Image shows demonstrative bands of the analyzed proteins in the livers. Bars represent the ratio of phosphorylated AMPKα to total AMPKα. HFFr: High-Fat, High-Fructose diet, S: Saline, +Met25: HFFr+25 mg/kg body weight metformin, +Met200: HFFr+200 mg/kg body weight metformin, +CaD: HFFr+2.4% Calcium plus 10000 IU/kg vitamin D_3_, *AMPK: AMP-activated protein kinase.* Data are mean ± SE. n = 6–7 in each group. There were no significant differences between groups (P>0.05).

There was no statistical difference between the seven groups with respect to the hepatic phosphorylation of AMPK (p-value>0.05) (Figure 4).

The suggested key role of AMPK in mechanism of *metformin action* prompted us to examine the involvement of AMPK in the observed findings. Here, we demonstrated that the phosphorylation of AMPK was not affected by neither HFFr diet nor different doses of metformin and/or calcium-vitamin D_3_. These findings are discordant from a large body of literature, which suggests that a high-fat diet exposure could decrease hepatic p-AMPK and metformin and calcitriol are activators of this intracellular energy sensor.^19,44,58,59^ In contrast, and in agreement with our findings, Foretz *et al.*^60^ reported that mice lacking hepatic AMPK exhibited normal blood glucose levels and gluconeogenic gene expression compared to those in wild-type mice‏. The maintenance of the antidiabetic effects of metformin in these animals as well as the reduction of intracellular ATP content clearly revealed that metformin suppressed gluconeogenesis independently of the liver-kinase B1 (LKB1)/AMPK pathway and via lowering hepatic energy status. This finding has been subsequently confirmed by other *investigators,*^35,36,61^ who disclosed the AMPK-independent effects of metformin in the management of type 2 diabetes and NAFLD, and suggested that metformin could ameliorate hepatic steatosis through silent mating type information regulation 2 homolog1 (SIRT1) -mediated effects on the autophagy machinery. In this study, we did not assess inflammatory pathways and fatty acid metabolism-related gene expressions, which could provide a direction for future research.

## Conclusion

In conclusion*, our results suggest that both* metformin and calcium-vitamin D_3_ provide similar hepatoprotective effects in an insulin and AMPK-independent manner, with slightly additional protective benefits of their combination on the steatosis scores and hepatic cholesterol content. *Ultimately,* the increased CTRP3 levels after metformin administration was also observed in the present study.

## Acknowledgments

The authors would like to express their gratitude towards Dr. Amir Abbas Farshid and Dr. Ali Asghar Tehrani for conducting the pathological assessments. The authors would also like to thank the Pharma chemie, Arya and Daana Pharmaceutical Companies and Beyza 21 Feed Mill Company. Financially, this study was supported by Urmia University of Medical Sciences.

## Ethical Issues

This study was conducted in accordance with the guide for care and use of laboratory animals approved by the Animal Ethical Committee of Urmia University of Medical Sciences.

## Conflict of Interest

The authors declare that they have no conflict of interest.
