# Erratum: Correction of Title

**DOI:** 10.34172/apb.2021.089

**Published:** 2020-11-07

**Authors:** Sara Shojaei Zarghani, Samin Abbaszadeh, Mohammad Alizadeh, Maryam Rameshrad, Alireza Garjani, Hamid Soraya

**Affiliations:** ^1^Student Research Committee, Urmia University of Medical Sciences, Urmia, Iran.; ^2^Cellular and Molecular Research Center, Department of Pharmacology, Faculty of Pharmacy, Urmia University of Medical Sciences, Urmia, Iran.; ^3^Department of Nutrition, Food and Beverages Safety Research Center, Faculty of Medicine, Urmia University of Medical Sciences, Urmia, Iran.; ^4^Department of Pharmacology and Toxicology, Faculty of Pharmacy, Tabriz University of Medical Sciences, Tabriz, Iran.

There was a misprint in the title of published article: The Eeffect of Metformin Combined with Calcium-Vitamin D3 Against Diet-Induced Nonalcoholic Fatty Liver Disease. Adv Pharm Bull, 2018, 8(1), 97-105. doi: 10.15171/apb.2018.012.^1^ In the title of this article, the word “Eeffect” was corrected to “Effect”.

The correct title of the article is:

The Effect of Metformin Combined with Calcium-Vitamin D3 Against Diet-Induced Nonalcoholic Fatty Liver Disease
